# A 3-year retrospective analysis of microbial species and key biomarkers associated with wound infections in Shantou Hospital, China

**DOI:** 10.3389/fcimb.2025.1549470

**Published:** 2025-05-19

**Authors:** Sabir Khan, Bing Hou, Muhammad Nadeem Khan, Muhammad Shafiq, Lin Cai, Wenjie Fang, Qian Wang, Hazrat Bilal, Yuebin Zeng

**Affiliations:** ^1^ Department of Dermatology, The Second Affiliated Hospital of Shantou University Medical College, Shantou, China; ^2^ Department of Clinical Laboratory, Skin and Venereal Diseases Prevention and Control Hospital of Shantou City, Shantou, Guangdong, China; ^3^ Department of Cell Biology & Genetics, Shantou University Medical College, Shantou, China; ^4^ Research Institute of Clinical Pharmacy, Shantou University Medical College, Shantou, China; ^5^ Department of Dermatology, Changzheng Hospital, Second Military Medical University, Shanghai, China; ^6^ Department of Medical-Surgical and Experimental Sciences University of Sassari Neurology Unit, Azienza Ospedaliera Universitaria (AOU), Sassari, Italy; ^7^ Jiangxi Key Laboratory of Oncology (2024SSY06041), Jiangxi Cancer Hospital and Institute, The Second Affiliated Hospital of Nanchang Medical College, Nanchang, Jiangxi, China; ^8^ Department of Dermatology, West China Second University Hospital, Sichuan University, Chengdu, Sichuan, China

**Keywords:** microorganisms, wound infections, biomarkers, antibiotics resistance, China

## Abstract

A biomarker is an important indicator of a normal physiological or pathological process, or a pharmacological response to a therapeutic intervention. This retrospective study aimed to measure blood biomarkers in wound patients, identify the microorganisms responsible for wound infections and determine their drug susceptibility patterns at a tertiary care hospital in China. The study was conducted between 2022 and 2024, including 279 patients. A total of 33 microbial species were isolated using culture techniques, identified, and analyzed for their antibiotic susceptibility. The organisms were predominantly gram-positive (50.8%), with *Staphylococcus aureus* (80.2%) being the most prevalent species. Among the gram-negative bacteria (41.2%), *Pseudomonas aeruginosa* (22.6%) was the most predominant species. Biomarkers such as white blood cells, neutrophils, lymphocytes, and erythrocyte sedimentation rate (ESR) values were higher than normal in most of microbial species associated with wound infections. The WBC value in gram-positive infections and the neutrophil and ESR values in fungal infections were statistically significantly higher than the normal range (*p* = 0.0002, *p* = 0.002, and *p* = 0.003, respectively). Albumin levels were high value in *P. aeruginosa* and *K. pneumoniae* (0.48 and 0.56 respectively), while lymphocytes levels were the lowest value (-0.62) in *S. aureus*. Resistance to at least one antibiotic was identified in 82.4% of the isolates. The prevalence of multidrug-resistant microbes in different wound infections is a significant concern in China. A health awareness campaign, coupled with improved hygiene measures, should be implemented to prevent the spread of microorganisms responsible for wound infections within the community.

## 1 Introduction

Skin serves as the first-line immune defense barrier against pathogen colonization. Therefore, alterations in its normal structure due to surgery or chemical, mechanical, or thermal trauma disrupt its function, leading to wounds (Prescott et al., 2017; Guan et al., 2021; Gowda et al., 2023). Regardless of the wound type, wound infections are frequently linked to patient morbidity and mortality, particularly in developing countries (Falanga et al., 2022). Treatment failure refers to increased healthcare expenses due to prolonged hospital stays for diagnostic testing, extensive antibiotic therapy, and in some cases of invasive surgery (Zuarez-Easton et al., 2017). The bacterial species most frequently responsible for wound infections include *A. baumannii*, *P. aeruginosa*, *S. aureus*, *K. pneumoniae*, and *E. faecalis* (Stevens, 2009; Ch’ng et al., 2019; Huszczynski et al., 2019; Puca et al., 2021). Some fungi have also been identified as causes wound infections. The entry of these organisms into the bloodstream and lymphatic system can trigger sepsis (Bilal et al., 2022).

A biomarker is measurable evidence that serves as an indicator of a normal physiological or pathological process, or a pharmacological response to a therapeutic intervention (Califf, 2018). Predictive biomarkers can predict outcomes or indicate the likelihood of therapy effectiveness. They may act as a powerful tool in tailoring therapeutic approaches for specific groups of patient populations. Diagnostic biomarkers can identify the presence of one or more factors that may influence clinical outcomes. An indicative biomarker can be utilized to determine disease development and/or therapeutic response in real time (Lindley et al., 2016).

The emergence of so-called “super-bugs,” or bacteria that are resistant to multiple drugs, is a significant public concern. Drug resistance results from the improper use of antibiotics in both humans and animals (Rahman et al., 2022). According to a 2014 WHO assessment, multidrug-resistant bacteria cause over 25,000 deaths annually in Europe and 23,000 deaths annually in the US (Diallo et al., 2020). Furthermore, approximately 50% of infections caused by *K. pneumoniae*, *S. aureus*, *P. aeruginosa*, and *E. coli* exhibit resistance to the most potent antibiotics, including third-generation cephalosporins (Diallo et al., 2020).

Large quantities of carbapenem-resistant, methicillin-resistant, and ESBL-producing bacteria have been found globally. The issue of ESBL producing microbes is particularly severe in developing countries (Iskandar et al., 2021). Therefore, bacteria that produce ESBL can resist the effects of these antibiotic classes (Nagshetty et al., 2021; Alfei and Schito, 2022). The most prevalent gram-negative bacteria producing ESBL include *E. coli*, *K. pneumoniae*, *P. mirabilis*, and *P. aeruginosa* (Oshiokhayamhe, 2023).

In this retrospective study, we identified the microbial species responsible for wound infections and analyzed their drug susceptibility patterns and determined the associated biomarker. The data provided can guide physicians in improving wound infection surveillance, prevention, and control.

## 2 Methods

### 2.1 Study site and population

This retrospective study was conducted at the Skin and Venereal Disease Prevention and Control Hospital located in Shantou City, Guangdong, China. The study period was 3 years, from January 2022 to September 2024. A total of 279 specimens (swabs, pus, tissue, and fluid) were collected from wounds by a trained nurse following standard procedures (Gjødsbøl et al., 2012). Patients with clinical signs of wound infection, such as redness, swelling, discomfort, persistent discharge, and an unpleasant odor were recorded (Gardner et al., 2001; Haesler et al., 2019). Data on patient type, age, gender, and infection sites were recorded in the wound care log database (Dong et al., 2019).

### 2.2 Routine laboratory protocol

#### 2.2.1 Sample collection

Pus specimens were collected from the patients wounds in hospital wards utilizing sterile cotton swabs and fine needle syringes. Samples were taken from various parts of the body, including leg, hand, back, abdomen, foot, breast, chest, head, and neck wounds. Each sample was labeled with the patient’s information, the collection approach, and the date and time of collection. Complete information about the patient was obtained, including the infection site, symptoms, and prior antibiotic therapy. Moreover, a complete blood count (CBC: leukocytes, neutrophils, and lymphocytes) was analyzed using an Automatic blood analyzer (Sysmex XN-1000 SA-01). Erythrocyte sedimentation rate (ESR) and albumin values were measured using a sediment detection frame and BS-800 Mandry biochemical instrument, respectively.

#### 2.2.2 Sample preparation, bacterial culture, and identification

Based on the type and location of the wound, specimens were visually assessed for consistency, color, turbidity, and the presence of blood. According to established clinical laboratory protocols, pus samples were inoculated onto blood agar, chocolate agar, MacConkey agar, nutrient agar, potato dextrose agar, Sabouraud dextrose agar, and CHROMagar-Candida medium. The VITEK 2 COMPACT system (bioMérieux, Marcy-l’Étoile, France) or the MALDI Biotyper system (Bruker Daltonics GmbH, Bremen, Germany) was used to identify the isolated bacteria and fungi according the manufacturer’s instructions.

#### 2.2.3 Analysis of the antimicrobial susceptibility patterns of the isolated organisms

Antimicrobial susceptibility testing was conducted using the VITEK 2 system (bioMérieux, Marcy-l’Étoile, France) according to the manufacturer’s guidelines. The tested antibiotics were used against bacterial and fungal isolates. The organisms were categorized as “susceptible,” “resistant,” or “intermediate,” according to the guidelines established by the (Clinical and Laboratory Standards Institute (CLSI), 2024) (CLSI M100-ED34: 2024 performance standards for antimicrobial susceptibility testing). Multidrug-resistant (MDR) strains were defined as isolates resistance to at least one antibiotic from two or more classes.

#### 2.2.4 Confirmation of MRSA and ESBL producers

Methicillin-resistant strains were suspected based on MIC results and confirmed using cefoxitin disc diffusion tests (Clinical and Laboratory Standards Institute (CLSI), 2007). ESBL-producing bacteria were detected among Enterobacteriaceae isolates using antibiotic discs that contained 30 μg of cefotaxime, ceftazidime, ceftriaxone, and aztreonam. According to standard guidelines, bacterial isolates with ceftazidime smaller than 22 mm and cefotaxime smaller than 27 mm were considered potential ESBL producers (Clinical and Laboratory Standards Institute (CLSI), 2007).

#### 2.2.5 Quality control and statistical analysis

A sterility examination was conducted on all produced biochemical medium and streaked plates. The reference strains *E. coli* ATCC 25922 and *S. aureus* ATCC 25923 were used as quality controls for antibacterial susceptibility testing and biochemical assays. The strains *C. albicans* ATCC 10231 and *C. parapsilosis* ATCC 22019 were used as quality controls for antifungal susceptibility testing. The DDST was also performed and phenotypically confirmed for ESBL-producing gram-negative bacilli using *E. coli* ATCC 25922 as a negative control.

Data analysis was conducted using GraphPad Prism version 8.0.2. Frequencies for categorical variables were calculated. The chi-square test was used for comparisons, with a *p*-value of less than 0.05 considered significant. Additionally, statistical analysis of the data was performed using R 4.4.1 software. Shapiro-Wilk tests were employed to determine if the groupings followed a normal distribution. Numerical data were presented as percentages and medians (25th–75th percentiles). R 4.4.1 along with respective libraries such as ggplot2, dplyr, tidyr, corrplot, and agricolae was used for analysis. ANOVA, followed by the *post-hoc* Tukey test, was applied to compare the impact of microorganisms on blood biomarkers. Pearson correlation was conducted to assess the associations between microorganisms and blood biomarkers. The results were presented as a heatmap.

## 3 Results

### 3.1 Clinical characteristics of patients with wound infections

**Figure 1:**
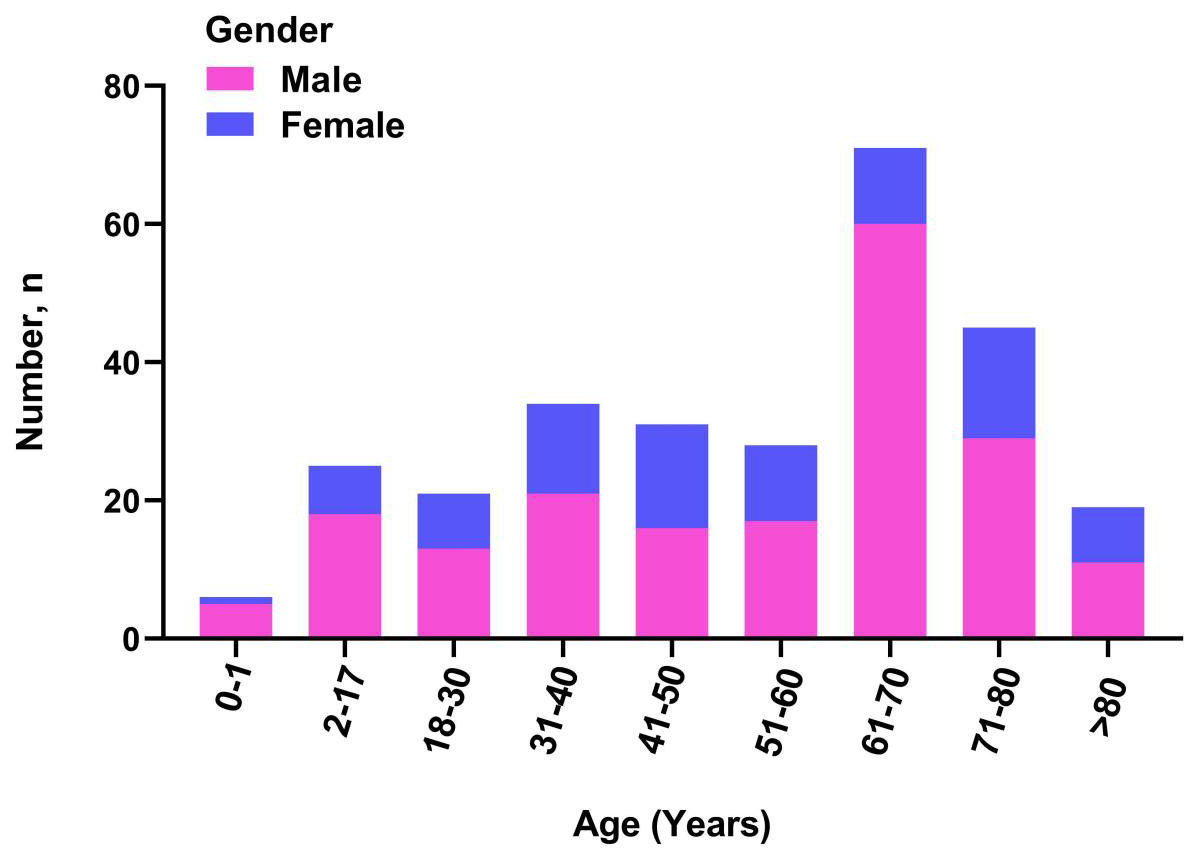
Distribution the number of wound cases among male and female.

Over the 3-year period, a total of 279 patients with clinical wound infections were monitored, including 90 (32.0%) females and 189 (68.0%) males. The distribution of wound infections in males and females is shown in **Figure 1**. The patients had a median age of 57 years (interquartile range: 38–68 years) and were analyzed between 2022 to 2024 (**Table 1**). A large number of patients were recruited from the inpatient department (I and II; 234 [84.0%]), followed by the dermatology outpatient department (45 [16.0%]).

**Table 1:** Characteristics of the sample separate by surveys years.

Characteristics	2022 N (%)	2023 N (%)	2024 N (%)	Total N (100%)
Sample	88 (31.5)	115 (41.2)	76 (27.2)	279 (100.0)
Gender				
Male	65 (34.3)	75 (40.0)	49 (25.9)	189 (68.0)
Female	23 (25.5)	40 (44.4)	27 (30.0)	90 (32.0)
Age, years median (IQ)	53.5 (38-66)	54 (34-68)	62(45-71)	57 (38-68)
Department				
Dermatology Inpatient Department 1, 2	56 (23.8)	102 (43.8)	76 (32.3)	234 (84.0)
Dermatology Outpatient Department	32 (71.1)	13 (28.8)	–	45 (16.0)

### 3.2 Analysis of microbial species and biomarkers

**Figure 2:**
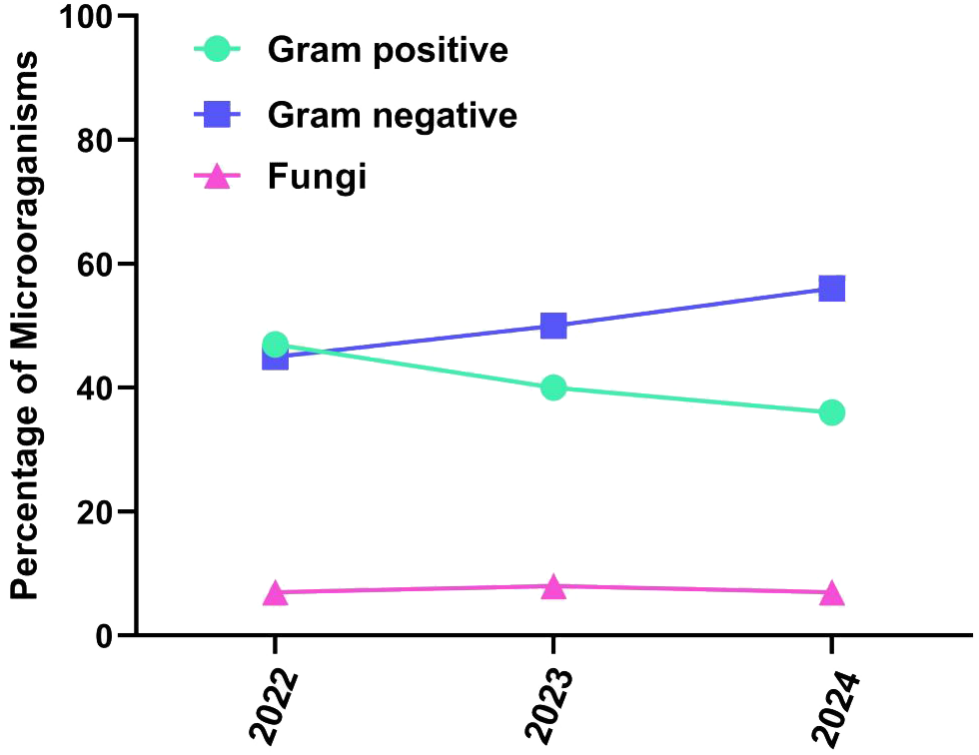
Trends of three years of microbial species causing wound infections.

**Figure 3:**
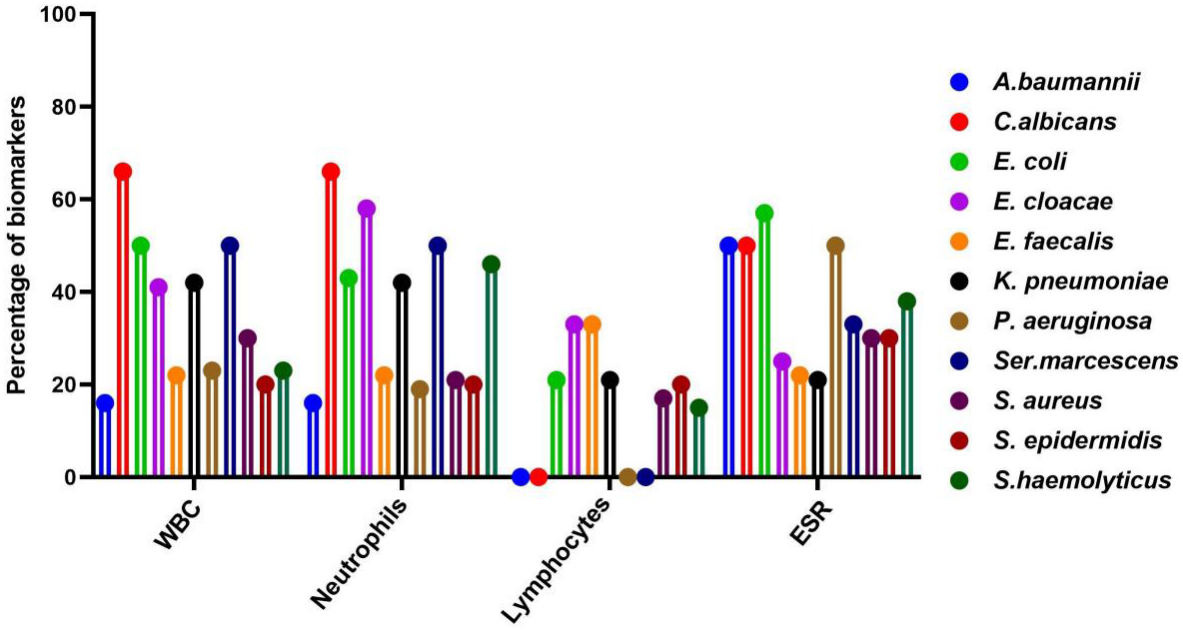
Distribution of microbial species associated with key biomarker abnormal range in wound infections. A, *Acinetobacter*; C, *Candida*; E. coli, *Escherichia Coli*; E, *Entercoccus*; K, *klebsiella*; P, *Pseudomonas*; and S, *staphylococcus*.

**Figure 4:**
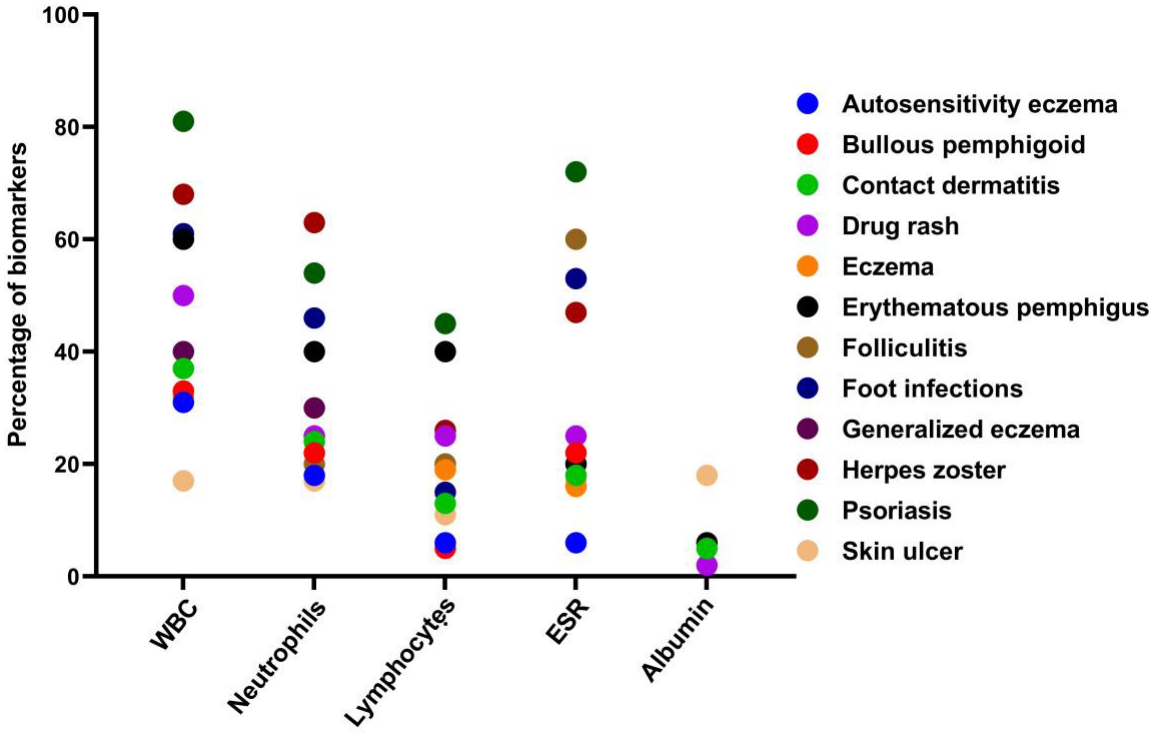
Percentage of abnormal value of key biomarker in patients with different skin infections.

**Figure 5:**
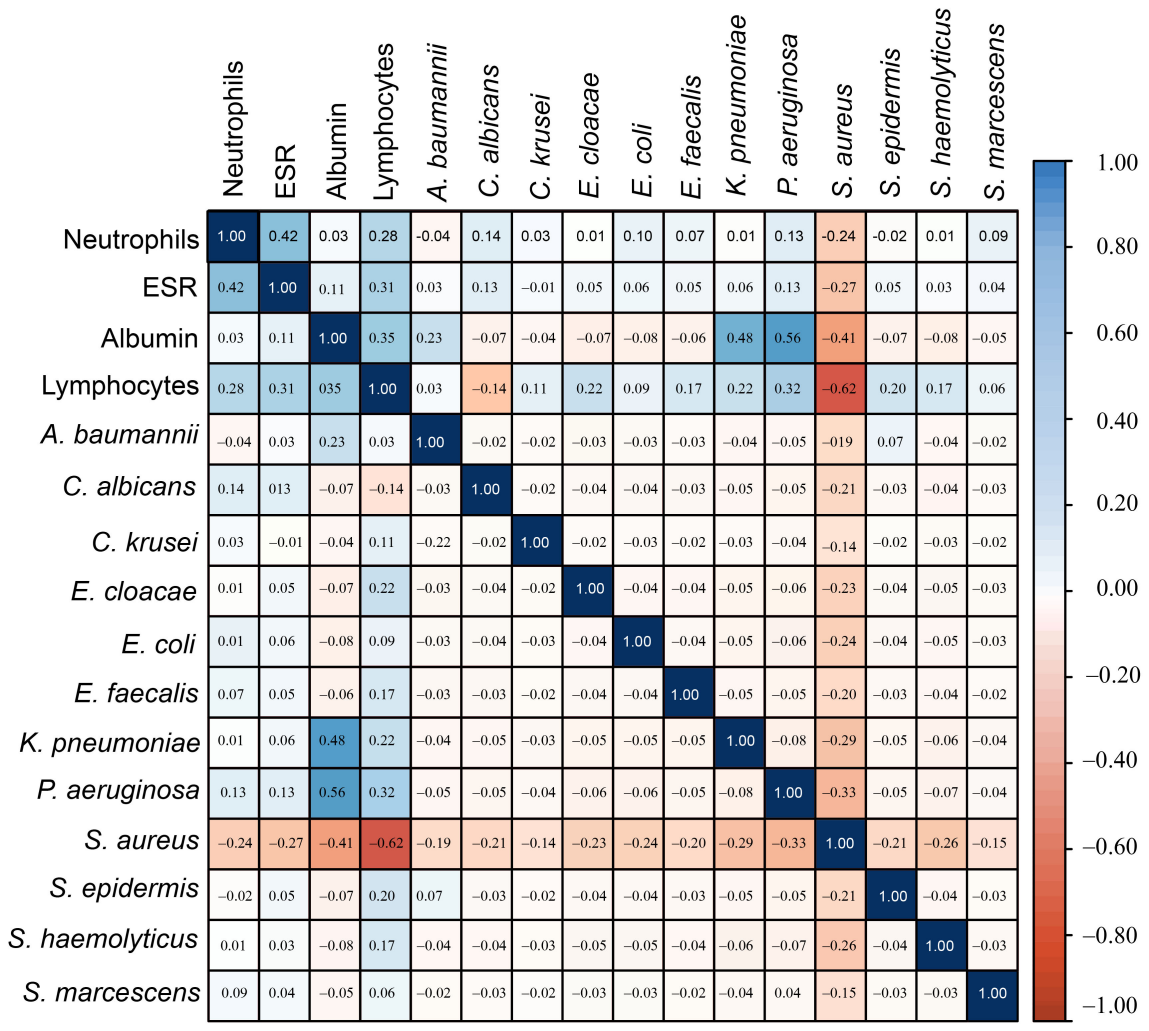
The correlation heatmap of microorganisms with Biomarkers showed that *S. aureus* had a strong correlation with lymphocytes, while *K. penumoniae* and *P. aeruginosa* showed a weak correlation with albumin.

A total of 279 microbial isolates were identified, of which 142 (50.8%) were gram-positive bacteria, 115 (41.2%) were gram-negative bacteria, and 20 (7.16%) were fungi. *S. aureus* was the most predominant (n = 114, 40.8%), followed by *P. aeruginosa* (n = 26, 9.31%), *K. pneumoniae* (n = 19, 6.81%), *E. coli* (n = 14, 5.01%), *S. haemolyticus* (n = 13, 4.65%), and *C. albicans* (n = 12, 4.30%). The distribution of microorganisms per year is shown in **Table 2** and **Figure 2**. A higher percentage of microbial isolates were identified in inpatients (n = 248, 89.5%) than in outpatients (n = 31, 10.4%) (**Supplementary Table S1**). The distribution of microbial species associated with biomarker in wound infections is shown in **Figure 3**. The leukocyte, neutrophil, and lymphocyte counts, as well as ESR values, of patients with gram-negative bacterial, gram-positive bacterial, and fungal infections were higher than the normal range (**Table 3**). The WBC value in gram-positive infections and neutrophil and ESR values in fungal infections were statistically significantly higher than the normal range (*p* = 0.0002, *p* = 0.002, and *p* = 0.003, respectively). The percentage of abnormal biomarkers leukocyte, neutrophil, and lymphocyte and ESR associated with different skin diseases is shown in **Figure 4** and **Table 4**. Moreover, the correlation between microorganisms and biomarkers is shown in **Figure 5**. Albumin shows a high value in *P. aeruginosa* and *K. pneumoniae*, while lymphocytes show the lowest value in *S. aureus*.

**Table 2:** Microorganisms distribution across survey years.

Species	2022 N (%)	2023 N (%)	2024 N (%)	Total N (100%)
**Gram Positive isolates**	**38 (45.2)**	**57 (50.8)**	**47 (56.6)**	**142 (50.8)**
*S.aureus*	25 (29.7)	46 (41.0)	41 (49.3)	114 (40.8)
*S.epidermidis*	2 (2.38)	6 (5.35)	2 (2.40)	10 (3.58)
*S.haemolyticus*	7 (8.33)	2 (1.78)	4 (4.81)	13 (4.65)
*S.hominis*	1 (1.19)	–	–	1 (0.35)
*Sh.ludgunensis*	2 (2.38)	3 (0.89)	–	5 (1.79)
*S.agalactiae*	1 (1.19)			1 (0.35)
**Gram negative isolates**	**40 (47.6)**	**45 (40.1)**	**30 (36.1)**	**115 (41.2)**
*A.baumannii*	1 (1.19)	2 (1.78)	3 (3.61)	6 (2.15)
*E.coli*	4 (4.76)	7 (6.25)	3 (3.61)	14 (5.01)
*Ecc.aerogenes*	1 (1.19)	–	–	1 (0.35)
*Ecc.cloacae*	4 (4.76)	3 (2.67)	5 (6.02)	12 (4.30)
*Ecc.faecalis*	3 (3.57)	5 (4.46)	1 (1.20)	9 (3.22)
*K.oxytoca*	2 (2.38)	1 (0.89)	1 (1.20)	4 (1.43)
*K.pneumoniae*	10 (11.9)	6 (5.35)	3 (3.61)	19 (6.81)
*N.gonorrhoeae*	4 (4.76)	2 (1.78)	3 (3.61)	9 (3.22)
*Morg.morganii*	–	2 (1.78)	–	2 (0.71)
*P.gergoviae*	–	2 (1.78)	–	2 (0.71)
*P. hauseri*	–	–	1 (1.20)	1 (0.35)
*P. mirabilis*	–	1 (0.89)	–	1 (0.35)
*P. aeruginosa*	7 (8.33)	13 (11.6)	6 (7.22)	26 (9.31)
*R.ornithinolytica*	–	1 (0.89)	–	1 (0.35)
*P.fluorescens*	1 (1.19)		1 (1.20)	2 (0.71)
*Ser.marcescens*	3 (3.57)	–	3 (3.61)	6 (2.15)
**Fungal isolates**	**6 (7.14)**	**10 (8.92)**	**4 (7.22)**	**20 (7.16)**
*C.albicans*	4 (4.76)	5 (4.46)	3 (3.61)	12 (4.30)
*C.krusei*	2 (2.38)	1 (0.89)	1 (1.20)	4 (1.43)
*C.parapsilosis*	–	1 (0.89)	–	1 (0.35)
*C.tropicalis*	–	3 (0.89)	–	3 (1.07)
**Total**	**84 (30.1)**	**112 (40.1)**	**83 (29.7)**	**279 (100)**

*S.*, Staphylococcus; *A.*, Acinetobacter; *E.*, Escherichia; *ECC*, Enterococcus; K, klebsiella; *N*, Neisseria; *Morg*, Morganella*; P, *Proteus*; R, * Raoultella*; P.fluorescens, Pseudomonas fluorescens*; *Ser*, Serratia; *Sh*, Shewanella; *C.*, Candida.

Bold values indicate the total number of microorganisms.

**Table 3:** Comparison of normal and abnormal range of laboratory measurements in patients with wound infections.

Mos	Biomarker	Normal range. Median (25th-75th)	Abnormal range. Median (25th-75th)	*P* value
Gram Negative	WBC, 10^3/^µL	8.515 (7.410 - 9.733)	14.09 (12.44 - 15.55)	0.328
	Neutrophils, 10^3/^µL	5.660 (5.080 - 6.175)	9.460 (7.340 - 11.74)	3.783
	Lymphocytes, 10^3/^µL	2.160 (1.635 - 2.565)	3.900 (3.403-4.673)	0.722
	ESR, mm/hour	8.500 (5.000 - 13.75)	50.00 (36.25 - 73.75)	1.353
Gram Positive	WBC, 10^3/^µL	9.180 (7.480 - 9.970)	13.49 (12.56 - 15.02)	**0.0002**
	Neutrophils, 10^3/^µL	5.620 (4.800 - 6.150)	9.410 (8.620 - 10.55)	3.059
	Lymphocytes, 10^3/^µL	1.800 (1.488 - 2.363)	5.245 (4.635 - 8.845)	0.924
	ESR, mm/hour	7.500 (5.000 - 13.00)	46.00 (31.50 - 67.50)	7.064
Fungi	WBC, 10^3/^µL	12.36 (9.170 - 12.93)	13.52 (12.85 - 16.11)	0.335
	Neutrophils, 10^3/^µL	8.130 (6.405 - 8.600)	9.090 (8.695 - 9.740)	**0.002**
	Lymphocytes, 10^3/^µL	–	–	–
	ESR, mm/hour	9.000 (5.000 - 11.25)	47.00 (34.00 - 75.00)	**0.003**

WBC, White blood cell; µL, Microliter; ESR, Erythrocyte Sedimentation Rate; mm, millimeters. Bold values indicate statically significant.

**Table 4:** Abnormal value of key biomarker in patients with different skin infections.

Biomarker	WBC *n* (%)	Neutrophils *n* (%)	Lymphocytes *n* (%)	ESR *n* (%)	Albumi*n* n (%)
Diagnosis n total (*n=*)					
Auto sensitivity eczema (*n*=16)	5 (31.2)	3 (18.7)	1 (6.25)	1 (6.25)	–
Bullous pemphigoid (*n*=18)	6 (33.3)	4 (22.2)	1 (5.55)	4 (22.2)	1 (5.55)
Contact dermatitis (*n*=37)	14 (37.8)	9 (24.3)	5 (13.5)	7 (18.9)	1 (2.70)
Drug rash (*n*=4)	2 (50.0)	1 (25.0)	1 (25.0)	1 (25.0)	–
Eczema (*n*=73)	24 (32.8)	16 (22.0)	14 (19.1)	12 (16.4)	5 (6.84)
Erythematous pemphigus (*n*=5)	3 (60.0)	2 (40.0)	2 (40.0)	1 (20.0)	–
Folliculitis (*n*=5)	3 (60.0)	(20.0)	(20.0)	3 (60.0)	–
Foot infections (*n*=13)	8 (61.5)	6 (46.1)	2 (15.3)	7 (53.8)	–
Generalized eczema (*n*=10)	4 (40.0)	3 (30.0)	2 (20.0)	2 (20.0)	–
Herpes zoster (*n*=19)	13 (68.4)	12 (63.1)	5 (26.3)	9 (47.3)	–
Psoriasis (*n*=11)	9 (81.8)	6 (54.5)	5 (45.4)	8 (72.7)	2 (18.1)
Skin ulcer (*n*=17)	3 (17.6)	3 (17.6)	2 (11.7)	3 (17.6)	–

### 3.3 Biomarkers associated with gram -negative bacteria

The prevalence of gram-negative and key biomarkers is shown in the **Supplementary Table S2**. *E. coli* and *Ser. marcescens* exhibited a high percentage (50.0%) of abnormal WBC ratios compared to normal range. Similarly, the percentage of abnormal neutrophil ratios compared to normal range was high in *K. pneumoniae* and *Ser. marcescens.* The abnormal value of lymphocytes compared with normal value was high in *E. faecalis*. Moreover, in *E. coli* and *P. aeruginosa* exhibited a high percentage of abnormal ESR ratios compared to the normal value. There was no statistically significant correlation between gram-negative bacteria and biomarkers such as WBC and ESR. While the correlation of neutrophils in *E. cloacae* and lymphocytes in *E. coli, E. cloacae* were identified statically significant (*p* < 0.05). Moreover, the correlation of *A.baumannii* with albumin was statistically significant (*p* < 0.05).

### 3.4 Biomarkers associated with gram- positive bacteria

The prevalence of gram-positive bacteria and key biomarkers is shown in the **Supplementary Table S3**. In *S.aureus*, the percentage (28.0%) of abnormal ratio of WBC]was high compared to the normal ratio. In *S. haemolyticus*, abnormal neutrophils value were detected at higher levels compared to the normal value. Similarly, the high percentage (20.0%) of abnormal lymphocytes values of compared to the normal range was found in *S. epidermidis*. Moreover, the abnormal ESR values were high in the *S.haemolyticus* and *S.ludgunensis*. There was no statistically significant correlation between gram-positive bacteria and biomarkers such as albumin, WBC, and ESR. However, the correlation of neutrophils in *S. aureus* was statistically significant (*p* < 0.05). Similarly, the correlation of lymphocytes was statistically significant (*p* < 0.05) in *S. aureus* and *S. epidermidis*.

### 3.5 Biomarkers associated with fungi

The distribution of fungi and key biomarkers is shown in the **Supplementary Table S4**. The high percentage (66.6%) of abnormal values of WBC and neutrophils compared with normal value were recorded in *C.albicans* and *C.tropicalis*. Similarly, a high percentage (50.0%) of abnormal lymphocyte values, compared with normal range, were found in *C. albicans*. Furthermore, a high percentage (33.3%) of abnormal lymphocyte values, compared with normal value were detected in *C.tropicalis*. There was no statistically significant correlation between fungi and biomarkers such as WBC, neutrophils, lymphocytes, ESR and albumins.

### 3.6 Antibiotic resistance patterns in wound samples from 2022 to 2024

The antibiotic resistance patterns showed that 82.4% of wound samples contained organisms resistant to at least one agent (**Table 5**). Of these samples, 18.6% showed resistance to only one antibiotic, 21.9% to two, 17.2% to three, 10.7% to four, 11.2% to five, and 20.0% to at least six. The assessment of resistance distribution, classified by survey year, indicated an increase in resistance from 2022 to 2024 (**Table 5**).

**Table 5:** Resistance profile per surveys year.

Resistance Profile	2022 N (%)	2023 N (%)	2024 N (%)	Total N (100%)
no	15 (32.6)	13 (28.2)	18 (39.1)	46 (17.6)
yes	68 (31.7)	70 (32.7)	76 (35.5)	214 (82.4)
Multi-resistance				
no	14 (35.0)	13 (32.5)	13 (32.5)	40 (18.6)
2 antimicrobials	11 (23.4)	16 (34.0)	20 (42.5)	47 (21.9)
3 antimicrobials	11 (29.7)	12 (32.4)	14 (37.8)	37 (17.2)
4 antimicrobials	6 (26.0)	8 (34.7)	9 (39.1)	23 (10.7)
5 antimicrobials	9 (37.5)	10 (41.6)	5 (20.8)	24 (11.2)
*≥*6 antimicrobials	17 (39.5)	11 (25.5)	15 (34.8)	43 (20.0)

### 3.7 Antibacterial susceptibility

*A. baumannii* showed 100% susceptibility to amikacin, co-trimoxazole, rifampin, and tigecycline, while *E. coli* demonstrated 100% susceptibility to vancomycin and oxacillin. In contrast, all *E. coli* isolates were resistant to 29 tested antibiotics, with resistance rates ranging from 11.1% to 100.0%. Similarly, all *E. cloacae* isolates were 100% susceptible to ceftriaxone, clotrimazole, and piperacillin/tazobactam. However, *E. cloacae* showed high resistance to voriconazole (75.0%). *K. pneumoniae* exhibited high susceptibility to ertapenem (87.5%), imipenem (84.6%), and piperacillin/tazobactam (84.6%), but demonstrated 100% resistance to tetracycline. *P. aeruginosa* isolates were highly susceptible to moxifloxacin and tigecycline (77.7%). In contrast, all *P. aeruginosa* isolates were resistant to 18 tested antibiotics, with values ranging from 20.0% to 50.0%. Moreover, seven *E. coli* isolates (53.8%), six *K. pneumoniae* isolates (46.1%), and one *K. oxytoca* isolates (25.0%) tested positive for ESBL. Among the gram-negative MDR isolates, *A. baumannii* (33.3%), *E. coli* (78.5%), *E. faecalis* (16.6%), *E. cloacae* (60.0%), *K. pneumoniae* (47.3%), and *P. aeruginosa* (8.69%) were identified.

*E. faecalis* isolates exhibited highly resistant to gentamicin (80%), tetracycline (75.0%), co-trimoxazole (66.6%), and erythromycin (62.5%). All isolates of *S. aureus* were sensitive to 28 tested antibiotics, with susceptibility rates ranging from 51.7% to 95.0%. All staphylococcal isolates, including *S. epidermidis* and *S. haemolyticus*, were 100% susceptible to ceftriaxone, cefazolin, tigecycline, and vancomycin. In contrast, *S. epidermidis* and *S. haemolyticus* showed 100% resistance to ampicillin and oxacillin. Among the total strains, 45.5% were MRSA strains, and 50.0% were resistant to ceftazidime, followed by penicillin (43.3%), oxacillin (41.2%), clindamycin (39.3%), itraconazole (33.3%), and cefoxitin (32.8%). *S. epidermidis* and *S. haemolyticus* showed significant resistance to various antibiotics, confirming their classification as methicillin-resistant coagulase-negative staphylococci. *S. epidermidis* exhibited 100% resistance to oxacillin, ceftriaxone, and cefazolin and 85.71% resistance to penicillin. *S. haemolyticus* showed 100% resistance to oxacillin, penicillin, ceftriaxone, cefazolin, vancomycin, and tetracycline. Additionally, *S. haemolyticus* showed the highest proportion (100%) of gram-positive MDR isolates, followed by *S. aureus* (70.5%) and *S. epidermidis* (70.0%).

### 3.8 Antifungal susceptibility

*C. albicans* isolates were 100% susceptible to amikacin. All *C. albicans* isolates showed susceptibility to six other tested antibiotics, with values ranging from 50.0% to 91.6%. *C. krusei* isolates were 100% susceptible to six tested antibiotics and 50.0% susceptible to the only tested fluconazole. *C. tropicalis* isolates were 100% susceptible to five tested antibiotics but resistant to imipenem (100%) and fluconazole (33.3%). Among the fungal MDR isolates, *C. tropicalis* (33.3%) and *C. albicans* (8.33%) were identified.

## 4 Discussion

CBC indices, specifically leukocytes, neutrophils, and lymphocytes, are important inflammatory markers and have been gaining increasing attention. They are usually considered indicators of subclinical inflammation. Their immediate availability offers a significant advantage (Serban et al., 2024). Furthermore, inflammatory indicators are believed to be correlated with the prognosis of bacterial infections. ESR is also commonly used to assess the presence and severity of several infections in patients with wounds (Chen et al., 2024; Mahmoud et al., 2024). In our study, WBC values were significantly higher than the normal range in gram-positive bacterial infections (*p* = 0.0002). Similarly, neutrophil and ESR values were significantly higher than the normal range in fungal infections (*p* = 0.0002 and *p* = 0.003, respectively). However, no significant differences were found in the WBC, neutrophils, ESR, and lymphocytes ratios between normal and abnormal ranges across in microbial species, as shown in **Table 4**. The correlation of neutrophils in *E. cloacae*, and *S. aureus* was statistically significant (*p* < 0.05). Similarly, the correlation of lymphocytes was statistically significant (*p* < 0.05) in *E. coli*, *E. cloacae*, *S. aureus* and *S. epidermidis.* Moreover, the correlation of *A.baumannii* with albumin was statistically significant (*p* < 0.05). Albumin shows a high value in *P. aeruginosa* and *K. pneumoniae*, while lymphocytes show the lowest value in *S. aureus*. The results showed that albumin value increases beyond the normal range, the chances of skin infections caused by *P. aeruginosa* and *K. pneumoniae* may be increase. Similarly, the skin infections caused by *S. aureus* are more likely when lymphocytes decrease (**Figure 5**). The microorganisms showed strong correlation with biomarkers in wound infections (Siddiqui and Bernstein, 2010). The neutrophils and lymphocytes as an indicator of systemic inflammation in bacterial infections, including wound infections caused by bacteria (Rigby and Deleo, 2012). However, further research is needed to study the correlation between biomarkers and microorganisms at the molecular level. In a wound infection, the immune system involves both innate and adaptive pathways, including white blood cells, lymphocytes, and changes in ESR rate (Strbo et al., 2014; Zahorec, 2021). Neutrophils, as first responders, execute phagocytosis and secrete enzymes to eliminate infections. Inflammatory signals trigger the production of acute-phase proteins such as fibrinogen, leading to an elevated ESR as an indicator of inflammation (Gulhar et al., 2018). Adaptive immunity activates with B cells producing antibodies to neutralize infections and enhance phagocytosis, while T cells regulate the immune response and eradicate infected cells (Bonilla and Oettgen, 2010). In wound infections, the typical abnormal ratio showed changes such as elevated WBC count (>11,000 cells/µL), increased neutrophils (>70% of WBC), and decreased lymphocytes (<20% of WBC). Additionally, ESR is also elevated (>20 mm/hr, often >50 mm/hr), indicating inflammation.

Routine testing in microbiology laboratories mainly depends on culture methods to identify and isolate suspected infections from swabs, pus, or tissue biopsies. This helps in species identification and antibiotic susceptibility determination to guide treatment. A standardized method support global surveillance to mitigate the rising incidence of antibiotic resistance (Maillard et al., 2021). This retrospective analysis identified 26 microbial species from infected wounds. Gram-positive bacteria constituted a high proportion of cases (50.8%) compared to gram-negative bacteria (41.2%), aligning with previous studies (Lakhey and Bhatt, 2007; Guan et al., 2021). However, numerous previous studies have reported a higher incidence of gram-negative isolates compared to gram-positive bacteria (Du et al., 2022). This variation in results may be attributed to differences in patients’ demographic characteristics (Ghosh et al., 2022).

Our findings were consistent with other studies, which have reporting that *S. aureus* was predominant and highly resistant to antibiotics. *S. aureus* usually produces biofilms in chronic wounds, resulting in drug resistance (Parastan et al., 2020). A total of 45.5% MRSA strains were identified in this study. Our findings revealed a significant increase in the prevalence of MRSA compared to a previous survey by Haonan Guan et al (Guan et al., 2021), which reported MRSA strains resistant to vancomycin. This highlights a concerning rise in the incidence of MRSA in China. *S. aureus* exhibited sensitivity to oxacillin at a rate of 58.8%, but 41.18% of the variant resistant to oxacillin. This figure was lower than that reported by Haonan Guan et al. in China (Guan et al., 2021). The differences might be attributed to variations in bacterial culture methods and the geographical location of the studies. MRSA resistance to vancomycin due to genetic acquisition (vanA/B), adaptive mutations Vancomycin-Intermediate *S. aureus*, or biofilm-mediated tolerance (Chang et al., 2003; Holmes et al., 2012; Alav et al., 2018).

The predominant gram-negative bacteria were *P. aeruginosa* and *E. coli*, as previously reported (Guan et al., 2021; Wang et al., 2024). These bacterial species interfere with wound healing (Serra et al., 2015; Wong et al., 2015). In this study, *P. aeruginosa* constituted 6.81% of the most prevalent gram-negative bacteria in chronic wounds. *P. aeruginosa* (14.8–16.7%) was have also been reported as the predominant gram-negative bacterium in chronic wounds in earlier studies (Gadepalli et al., 2006; Wong et al., 2015). Another study demonstrated that *P. aeruginosa* was associated with deeper tissue layer invasion (Fazli et al., 2009). In our study, we also found that *P. aeruginosa* was highly resistant to antibiotics such as erythromycin, gentamicin, nitrofurantoin, and rifampin (> 30%) but showed at least > 25% resistance to vancomycin, moxifloxacin, and tigecycline. Additionally, *A. baumannii* demonstrated to 100% susceptibility to amikacin and tigecycline. The high susceptibility of *A. baumannii* to tigecycline (96.1%) and amikacin (96.4%) was also previously observed (Alhussain et al., 2021). Amikacin and tigecycline may experience minimal resistance development due to their efficient mechanism of action, limited misuse, resistance to prevalent strains, and their role in effective antimicrobial management within the study setting. Imipenem showed resistance rates below 20% for *E. coli* and *K. pneumoniae*; however, resistance increased in *E. cloacae*. Amikacin also showed resistance rates below 20% for *E. coli* and *E. cloacae*. Gram-negative bacteria have lowered resistance to amikacin, consistent with previously reported (Guan et al., 2021). *P. aeruginosa*, *E. coli*, and *A. baumannii* acquire carbapenem resistance mainly due to *bla*_NDM_, *bla*_KPC_, and OXA-type carbapenemases. Efflux pumps such as, mexAB-oprM in *P. aeruginosa*, adeABC in *A. baumannii* enhance multidrug resistance, while *E. coli* frequently developed plasmid-borne resistance genes like *mcr-1* and *blaCTX*-*M*-15 (Alav et al., 2018; Eichenberger and Thaden, 2019; Hammoudi Halat and Ayoub Moubareck, 2020; Gauba and Rahman, 2023).

*Klebsiella* spp. developed significant resistance to ß-lactam antibiotics, consistent with previous studies conducted in China (Chong et al., 2018). Similarly, a review of antimicrobial resistance trends in Asia highlighted the growing threat of multidrug-resistant *Klebsiella* spp (Effah et al., 2020). *K. pneumoniae* resists β-lactams due to ESBLs (CTX-M SHV), carbapenemases (KPC and NDM), and AmpC enzymes (DHA and CMY) (Tooke et al., 2019; Yang et al., 2022). Antimicrobial resistance due to antibiotic overuse is a critical global health threat, affecting individuals of all genders and ages equally (Effah et al., 2020). The resistance of *C. albicans* ranged from 8.33% to 50.0%. Thus, *C. albicans* demonstrated a high level of antibiotic resistance, aligning with previous reports (Bilal et al., 2022).

The prevalence of the MDR profile was higher in gram-positive bacteria compared to gram-negative bacteria. The MDR rate for gram-positive bacteria was 50.3%, which aligns closely with the rates reported in previous studies in Egypt (Ahmed et al., 2023), but different from study reported in China (Guan et al., 2021). In contrast, gram-negative isolates had an overall MDR rate of 37.6%, with the highest MDR profile found in *E. coli* (34.3%), followed by *K. pneumoniae* (28.1%) and *E. faecalis* (7.05%). These results were lower than the findings reported by Haonan Guan et al (Guan et al., 2021). Regarding the MDR profiles of fungi, *C. albicans* and *C. tropicalis* had the lowest MDR rates (1.72%). Similar findings have been previously reported (Puca et al., 2021). However, the variation may be due to regional antibiotic prescribing practices, prolonged hospital stays enhancing exposure to nosocomial pathogens, or the lack of robust antimicrobial stewardship programs in our hospital setting. The absence of molecular characterization restricts our study from tracking transmission pathways or confirming clonal outbreaks.

The division of isolates by year is another important aspect of this study. Particularly, in 2022, the percentages of isolated gram-negative and gram-positive bacteria were similar. However, in the subsequent years 2023 and 2024, there was an increase of gram-positive isolates (approximately 57%), along with a slight decrease in the number of gram-negative bacteria. In contrast, the proportion of fungal strains showed no significant variations over time, suggesting an increased spread of gram-positive species. The main limitations of this study are as follows: 1. The presence of anaerobic and microaerophilic bacteria was not detected; 2. No data on polymicrobial infections were collected; 3. The study findings should be applied with caution due to variations in individuals and circumstances across geographic locations. However, the findings of this study’s are important for developing strategies to improve the management of wound infections in our hospital and similar settings. The biomarker analysis integrating into clinical practice, clinician can achieve early detection, targeted treatments, better prevention, and infection control measures, as results lead to better patient outcomes and diminished complications in wound infections.

## 5 Conclusion

In this study, the most common species causing wound infections included *S. aureus* and *P. aeruginosa*. Among the gram negative, *E. coli* and *Ser. marcescens* showed a higher percentage of abnormal WBC ratios compared to the normal range. Among gram-positive bacteria, the percentage of *S.aureus* with abnormal WBC ratios was higher compared with normal range. However, WBC values were significantly higher than the normal range in gram-positive bacterial infections (*p* = 0.0002). While neutrophils and ESR values were significantly higher than the normal range in fungal infections (*p* = 0.0002 and *p* = 0.003, respectively. Albumin shows a high value in *P. aeruginosa* and *K. pneumoniae* (0.48 and 0.56 respectively), while lymphocytes show the lowest value (-0.62) in *S. aureus*. The results indicated that as albumin value exceed the normal range, the chances of skin infections caused by *P. aeruginosa* and *K. pneumoniae* increases. Similarly, the skin infections caused by *S. aureus* also increase with a decrease in lymphocytes ratios. Overall, it is very important to further investigate the associated between key biomarkers and microbial species to effectively control and manage wound infections.

## Data Availability

The original contributions presented in the study are included in the article/**Supplementary Material**. Further inquiries can be directed to the corresponding authors.
