# Supplementary material for: A 3-year retrospective analysis of microbial species and key biomarkers associated with wound infections in Shantou Hospital, China
Source: Front Cell Infect Microbiol. 2025 May 19;15:1549470. doi: 10.3389/fcimb.2025.1549470 (PMC12127373; doi:10.3389/fcimb.2025.1549470)
Supplement: Supplementary file 1 [file Table1.docx]

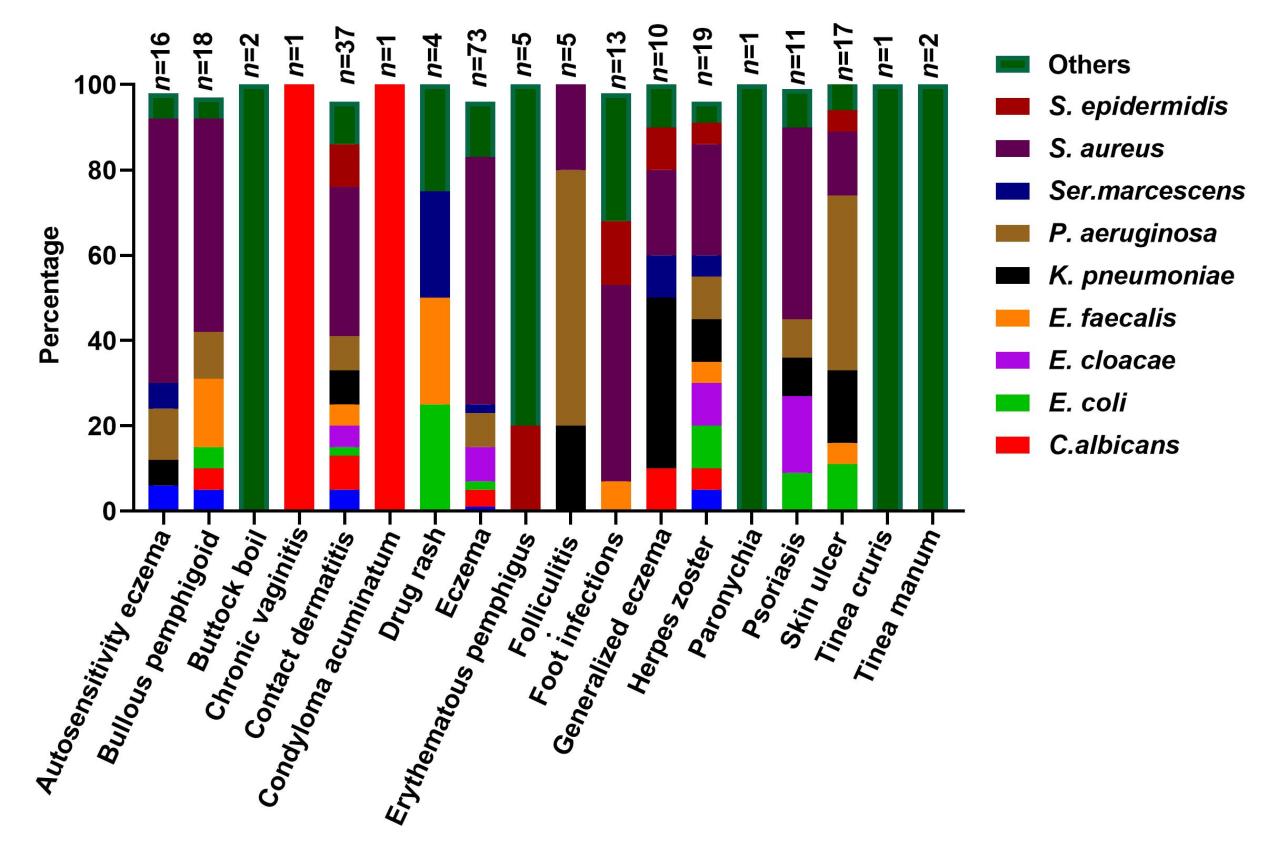


**Fig S1:** Distribution of microbial species causing different type of wound infections. Aci; Acinetobacter, E; Escherichia, Entero; Entercoccus, K; klebsiella, Ps; Pseudomonas, and S; staphylococcus.

**Table S1:** Bacterial isolates of skin among inpatients and outpatients attending the Skin and Venereal Diseases Prevention and Control Hospital of Shantou City.

| **Gram negative isolates** | **Inpatient *n* (%)** | **Outpatient *n* (%)** | **Frequency *n* (%)** |
| --- | --- | --- | --- |
| *Aci.baumannii* | 6 (2.41) | - | 6 (2.15) |
| *E.coli* | 14 (5.64) | - | 14 (6.09) |
| *Ent.aerogenes* | - | 1 (3.22) | 1 (0.35) |
| *Ent.cloacae* | 11 (4.43) | 1 (3.22) | 12 (4.30) |
| *Entero.faecalis* | 9 (3.62) | - | 9 (3.22) |
| *K.oxytoca* | 2 (0.80) | 2 (6.45) | 4 (1.43) |
| *K.pneum pneumoniae* | 16 (6.45) | 3 (9.67) | 19 (6.81) |
| *Neis.gonorrhoeae* | 9 (3.62) | - | 9 (3.22) |
| *Morg.morg.morganii* | 2 (0.80) | - | 2 (0.71) |
| *Plur.gergoviae* | 1 (0.40) | 1 (3.22) | 2 (0.71) |
| *Proteus hauseri* | 1 (0.40) | - | 1 (0.35) |
| *Proteus mirabilis* | 1 (0.40) | - | 1 (0.35) |
| *Ps.aeruginosa* | 21 (8.46) | 5 (16.1) | 26 (9.31) |
| *Raou.ornithinolytica* | 1 (0.40) | - | 1 (0.38) |
| *Ps.fluorescens* | 2 (0.80) | - | 2 (0.71) |
| *Ser.marcescens* | 6 (2.41) | - | 6 (2.15) |
| **Gram Positive isolates** |  |  |  |
| *Staph.aureus* | 104 (41.9) | 10 (32.2) | 114 (40.8) |
| *Staph.epidermidis* | 9 (3.62) | 1 (3.22) | 10 (3.58) |
| *Staph.haemolyticus* | 12 (4.83) | 1 (3.22) | 13 (4.65) |
| *Staph.hominis ssp homins* | 1 (0.40) | - | 1 (0.35) |
| *Staph.ludgunensis* | 4 (1.72) | 1 (3.22) | 5 (1.79) |
| *Str.agalactiae* | - | 1 (3.22) | 1 (0.35) |
| **Fungal isolates** |  |  |  |
| *C.albicans* | 10 (4.03) | 2 (6.45) | 12 (4.30) |
| *C.krusei* | 3 (1.20) | 1 (3.22) | 4 (1.43) |
| *C.parapsilosis* | 0 | 1 (3.22) | 1 (0.35) |
| *C.tropicalis* | 3 (1.20) | 0 | 3 (1.07) |
| **Total** | **248 (89.5)** | **31 (10.4)** | **279 (100)** |

**Table S2:** Biomarker analysis of gram-negative bacterial isolates.

| **Mos** | ****Biomarker**** | ****Below Normal *n* (%)**** | ****Normal****  *****n* (%)**** | ****Above Normal *n* (%)**** | *****P* value**** |
| --- | --- | --- | --- | --- | --- |
| ***A.baumannii*** | WBC | - | 5 (83.3) | 1 (16.6) |  |
|  | Neutrophils | - | 5 (83.3) | 1 (16.6) |  |
|  | Lymphocytes | - | - | - |  |
|  | ESR | - | 3 (50.0) | 3 (50.0) | ***P* < 0.05** |
|  | Albumin(g/L) | 1 (16.6) | 5 (83.3) | - | ***P* < 0.05** |
|  |  |  |  |  |  |
| ***E.coli*** |  |  |  |  |  |
|  | WBC | - | 7 (50.0) | 7 (50.0) |  |
|  | Neutrophils | 1 (7.14) | 8 (57.1) | 5 (35.7) |  |
|  | Lymphocytes | - | 11 (78.5) | 3 (21.4) | ***P* < 0.05** |
|  | ESR | 1 (7.14) | 6 (42.8) | 7 (50.0) |  |
|  | Albumin(g/L) | - | - | - |  |
|  |  |  |  |  |  |
| ***E.cloacae*** |  |  |  |  |  |
|  | WBC | 1 (8.33) | 7 (58.3) | 4 (33.3) |  |
|  | Neutrophils | 2 (16.6) | 5 (41.6) | 5 (41.6) | ***P* < 0.05** |
|  | Lymphocytes | 2 (16.6) | 8 (66.6) | 2 (16.6) | ***P* < 0.05** |
|  | ESR | - | 9 (75.0) | 3 (25.0) |  |
|  | Albumin(g/L) | - | - | - |  |
|  |  |  |  |  |  |
| ***E.faecalis*** |  |  |  |  |  |
|  | WBC | - | 7 (77.7) | 2 (22.2) |  |
|  | Neutrophils | - | 7 (77.7) | 2 (22.2) |  |
|  | Lymphocytes | - | 6 (66.6) | 3 (33.3) |  |
|  | ESR | - | 7 (77.7) | 2 (22.2) |  |
|  | Albumin(g/L) | - |  |  |  |
|  |  |  |  |  |  |
| ***K.oxytoca*** |  |  |  |  |  |
|  | WBC | - | 3 (75.0) | 1 (25.0) |  |
|  | Neutrophils | - | 3 (75.0) | 1 (25.0) |  |
|  | Lymphocytes | - | - | - |  |
|  | ESR | - | - | - |  |
|  | Albumin(g/L) | - | - | - |  |
|  |  |  |  |  |  |
| ***K.pneumoniae*** |  |  |  |  |  |
|  | WBC | - | 11 (57.8) | 8 (42.1) |  |
|  | Neutrophils | - | 11 (57.8) | 8 (42.1) |  |
|  | Lymphocytes | 1 (5.26) | 15 (78.9) | 3 (15.7) |  |
|  | ESR | - | 15 (78.9) | 4 (21.0) |  |
|  | Albumin(g/L) | 1 (5.26) | 17 (89.4) | 1 (5.26) |  |
|  |  |  |  |  |  |
| ***P. aeruginosa*** |  |  |  |  |  |
|  | WBC | 1 (3.84) | 20 (76.9) | 5 (19.2) |  |
|  | Neutrophils | 1 (3.84) | 21 (80.7) | 4 (15.3) |  |
|  | Lymphocytes | - | - | - |  |
|  | ESR | - | 13 (50.0) | 13 (50.0) |  |
|  | Albumin(g/L) | 5 (19.2) | 21 (80.7) | - |  |
|  |  |  |  |  |  |
| ***Ser. marcescens*** |  |  |  |  |  |
|  | WBC | - | 3 (50.0) | 3 (50.0) |  |
|  | Neutrophils | - | 3 (50.0) | 3 (50.0) |  |
|  | Lymphocytes | - | - |  |  |
|  | ESR | - | 4 (66.6) | 2 (33.3) |  |
|  | Albumin(g/L) | - | - | - |  |

**Table S3:** Biomarker analysis of gram-positive bacterial isolates.

| **Mos** | ****Biomarker**** | ****Below Normal (n) %**** | ****Normal (n)**** | ****Above Normal (n)**** | *****P* value**** |
| --- | --- | --- | --- | --- | --- |
| ***S. aureus*** |  |  |  | 114 |  |
|  | WBC | 3 (2.63) | 79 (69.2) | 32 (28.0) |  |
|  | Neutrophils | 1 (0.87) | 89 (78.0) | 24 (21.0) | ***P* < 0.05** |
|  | Lymphocytes | 7 (6.14) | 94 (82.4) | 13 (11.4) | ***P* < 0.05** |
|  | ESR | 13 (11.4) | 79 (69.2) | 22 (19.2) |  |
|  | Albumin(g/L) |  |  |  |  |
|  |  |  |  |  |  |
| ***S.epidermidis*** |  |  |  | 10 |  |
|  | WBC | - | 8 (80.0) | 2 (20.0) |  |
|  | Neutrophils | - | 8 (80.0) | 2 (20.0) |  |
|  | Lymphocytes | - | 8 (80.0) | 2 (20.0) | ***P* < 0.05** |
|  | ESR | 2 (20.0) | 7 (70.0) | 1 (10.0) |  |
|  | Albumin(g/L) |  |  |  |  |
|  |  |  |  |  |  |
| ***S.haemolyticus*** |  |  |  | 13 |  |
|  | WBC | - | 10 (76.9) | 3 (23.0) |  |
|  | Neutrophils | - | 7 (53.8) | 6 (46.1) |  |
|  | Lymphocytes | 1 (7.69) | 12 (92.3) | 1 (7.69) |  |
|  | ESR | - | 8 (61.5) | 5 (38.4) |  |
|  | Albumin(g/L) | - | - | - |  |
|  |  |  |  |  |  |
| ***S.ludgunensis*** |  |  |  |  |  |
|  | WBC | - | 3 (80.0) | 1 (20.0) |  |
|  | Neutrophils | - | - | - |  |
|  | Lymphocytes | - | - | - |  |
|  | ESR | - | 3 (80.0) | 1 (20.0) |  |
|  | Albumin(g/L) | - | - | - |  |

**Table S4:** Biomarker analysis of fungal isolates.

| **Mos** | ****Biomarker**** | ****Below Normal (n)**** | ****Normal (n)**** | ****Above Normal (n)**** | *****P* value**** |
| --- | --- | --- | --- | --- | --- |
| ***C.albicans*** | WBC | - | 4 (33.3) | 8 (66.6) |  |
|  | Neutrophils | - | 4 (33.3) | 8 (66.6) |  |
|  | Lymphocytes | - | - | - |  |
|  | ESR | - | 6 (50.0) | 6 (50.0) |  |
|  | Albumin(g/L) | - | - | - |  |
|  |  |  |  |  |  |
| ***C.krusei*** |  |  |  |  |  |
|  | WBC | - | 2 (50.0) | 2 (50.0) |  |
|  | Neutrophils | - | 2 (50.0) | 2 (50.0) |  |
|  | Lymphocytes | 1 (25.0) | 3 (75.0) | - |  |
|  | ESR | - | 3 (75.0) | 1 (25.0) |  |
|  | Albumin(g/L) | - | - | - |  |
|  |  |  |  |  |  |
| ***C.tropicalis*** |  |  |  |  |  |
|  | WBC | - | 1 (33.3) | 2 (66.6) |  |
|  | Neutrophils | - | 1 (33.3) | 2 (66.6) |  |
|  | Lymphocytes | - | 2 (66.6) | 1 (33.3) |  |
|  | ESR | - | 2 (66.6) | 1 (33.3) |  |
|  | Albumin(g/L) | - | - | - |  |
|  |  |  |  |  |  |
